# Programme Costing of a Physical Activity Programme in Primary Prevention: Should the Costs of Health Asset Assessment and Participatory Programme Development Count?

**DOI:** 10.1155/2012/601631

**Published:** 2012-03-25

**Authors:** Silke B. Wolfenstetter, Bernd Schweikert, Jürgen John

**Affiliations:** ^1^Helmholtz Zentrum München, German Research Center for Environmental Health, Institute of Health Economics and Health Care Management, Ingolstädter Land Straße 1, 85764 Neuherberg, Germany; ^2^Life Sciences, OptumInsight, Munich, Germany

## Abstract

This analysis aims to discuss the implications of the “health asset concept”, introduced by the WHO, and the “investment for health model” requiring a “participatory approach” of cooperative programme development applied on a physical activity programme for socially disadvantaged women and to demonstrate the related costing issues as well as the relevant decision context. The costs of programme implementation amounted to €48,700. Adding the costs for developing the programme design of €48,800 results in total costs of €97,500; adding on top of that the costs of asset assessment running to €35,600 would total €133,100. These four different cost figures match four different types of potentially relevant decisions contexts. Depending on the decision context the total costs, and hence the incremental cost-effectiveness ratio of a health promotion intervention, could differ considerably. Therefore, a detailed cost assessment and the identification of the decision context are of crucial importance.

## 1. Introduction

At the moment, more than half of the global population is not physically active to a satisfactory extent [1]. The increasing prevalence of physical inactivity has become an important public health problem worldwide, which has been suggested to be caused by various environmental as well as behavioural factors such as the rising use of transportation, increasing sedentary behaviour during work, and domestic activities or lack of sports and recreation facilities [2]. Physical inactivity is associated with many diseases such as obesity, coronary heart disease, diabetes mellitus type 2, osteoporosis, acute and chronic back pain as well as depression [2], and the risk-lowering positive health effects of regular physical activity have been substantiated in many reviews [3–12]. The negative health effects of physical inactivity lead to a rising economic burden to society, particularly as a result of increasing health care costs and productivity losses [13–15]. Savings due to physical activation of the population have been shown for different countries, for example, Switzerland, Austria, and USA [16–19].

The WHO proclaims the necessity of preventive efforts in policies and the environment aiming at promoting physical activity [2, 20]. Aspects of the accessibility of the target group and cross-linking as well as cooperation with health promoters are very important; these aspects are included in the “health assets concept” of health promotion introduced by the WHO [21–23]. In this concept, a health asset is any factor that enhances the ability of individuals, communities, populations, and/or social systems to improve or maintain health and well-being. This concept includes salutogenetic factors (“health assets”) that strengthen health in contrast to the prevention of illness [23] and promote possibilities for individuals and communities to be coproducers of health rather than simply consumers of health care services. Therefore, the objective of the health assets concept is to identify and mobilize available resources for health promotion that exist in the target population and their setting, for example, to provide the opportunity for leisure time physical activity or to improve accessibility to sport facilities for women in difficult life situations [21, 22]. There is evidence that in neighbourhoods with higher density of and proximity to sport facilities a higher level of physical activity can be found [24, 25]. In particular, socially disadvantaged women show a high prevalence of physical inactivity and of related diseases such as cardiovascular problems and diabetes mellitus [26, 27], and a need for promoting effective physical activity programmes in this population group has been stated by many observers [28]. The WHO approach is expected particularly to reduce socioeconomic inequalities in health. The “investment for health model” requires a “participatory approach” in cooperative programme development. The participation of this target group can be accomplished by building a cooperative planning group in the specific setting. This group could include project partners, representatives of the target group, and stakeholders from related policies that are conjointly involved in project planning, the implementation of structural changes, and the development of new physical activity programmes suited to the target population [29, 30]. There is still a lack of substantial research on health promotion with regard to successful activation of those target groups for whom more physical exercise would be particularly beneficial.

Moreover, the economic evaluation of those effective primary preventive physical activity programmes suited to a specific group becomes more and more important in a society with scarce resources. It should be based on the identification, measurement, and valuation of the costs and consequences of a prevention programme [31, 32]. The evaluation of primary prevention programmes is a complex challenge, and there is still debate on the criteria that are suitable to judge the performance of these programmes [32–34]. This discussion can be extended to the dimension of the costs and which costs have to be assessed and included in the economic evaluation according to the decision maker's context.

The objective of this analysis is three fold. First, we show that the costs of a health promotion programme may comprise more than simply the costs of running the programme. This is particularly true if the approach to developing and implementing appropriate programmes follows the health asset model proposed by the WHO, as this approach calls for resource-consuming activities aimed at identifying and mobilizing the health assets available in the target population and their setting as well as involving the target groups and other stakeholders in the design of the programme. Second, empirical data from a recent participatory research project are presented in order to illustrate that the inclusion of these additional cost elements may alter the results of programme costing and, in consequence, of cost-effectiveness analyses substantially. Third, we want to contribute to the discussion about whether and when the costs of health asset assessment and participatory project development should be included in programme costing to evaluate the cost-effectiveness of the programme under consideration.

## 2. Methods

### 2.1. Setting

This paper is based on data from a local neighbourhood project, which was conducted from January 2005 to December 2007. The development and evaluation of the primary preventive interventions was carried out in three different settings: “company,” “sports club,” and “residential district” in the area of Erlangen, Germany. The target population consisted of women “in difficult life situations” partly living in a residential district that was characterized by high rates of unemployment; social welfare recipients and migrants. The key characteristics of being “in a difficult life situation” included the receipt of low income or social welfare payments, low educational attainment, unemployed or in blue-collar occupation, and single parent or member of an ethnic minority. The project aimed to improve the opportunities for physical activity among this service population and define their interests at group meetings. Following the health assets concept and the participatory approach of project development, a setting group and a joint group were set up. The setting group mainly consisted of members of the target population, and the responsibilities within each setting included deciding on actions that should be taken for the promotion of physical activity among them; the joint group consisted of members of the target group, scientific experts, and setting-specific decision makers such as the management of the sports club; the mayor of Erlangen; other stakeholders and representatives of the company, who developed preventive exercise programmes suited to the target population and decided on instruments for evaluation in a cooperative work process. As these prevention programmes should be potentially transferable to other regions, the programmes were implemented and funded according to setting-specific arrangements.

The developed intervention programmes consisted of two different kinds of physical activity programmes, a 90-minute programme of moderate-to-high intensity (in the following called HI [high intensity] programme) and a 60-minute programme of low-to-moderate intensity (in the following called LI [low intensity] programme). Both programmes included a mix of different and from session-to-session changing elements of physical activity exercises such as endurance training, workout training, aerobic gymnastics, or relaxation exercises. The programmes took place as indoor programmes in gyms and once a week over a time period of three months, corresponding to a number of totally 11-12 sessions. The programmes were performed as group exercises, with a mean group size of 10–12 women. The exercise classes were directed by certified exercise instructors for grassroots sport and sport for all, being the first grade in the educational hierarchy of sport instructors and trainers and usually practised as a second-job activity. The acquisition of this certificate which is granted be the German public sport associations, is not restricted to an educational background in sport or healthcare; essentially, it requires the successful completion of a course consisting of regularly 120 hours of theoretical and practical training.

These two types of physical exercise programmes were offered in all three settings [22, 30, 35]. Recruitment to the project was done by applying usual marketing techniques such as production and distribution of flyers, holding informative meetings, or publishing newspaper articles on project activities. Moreover, and much more importantly, three different types of “social catalysts” [35] were effective in the process of recruiting women for the project. First, disadvantaged women themselves acted as social catalysts, leading to the involvement of other disadvantaged women in the implementation of the project. Second, informal social networks—particularly those of immigrant women—were especially helpful in initiating and sustaining women's participation and collaboration in project implementation. Third, a number of voluntary associations such as sports clubs, church communities, or cultural clubs of migrants which may be regarded as social institutions mediating between private and public life acted as social catalysts as well [35].

Totally, 87 women could be recruited for participation in the physical exercise programmes. Recruitment to these programmes was not linked to the obligation to participate in the evaluation research activities of the project. In fact, only 57 women were willing to participate in these research activities. Moreover, recruitment to the programmes as well as to other project activities was done on a self-selection basis. Therefore, women not being in difficult life situations had the option to participate. However, survey results showed that more than 90 percent of the 57 study participants were women in difficult life situations as defined by the project. As the study should produce realistic estimates of the willingness to participate in health promotion activities, no financial incentives were offered to the women for acting as study participants or participating in the physical activity programmes. Quite the contrary, the programmes were offered as programmes liable to charges, even if the level of the fees was fixed somewhat below the customary prices of comparable programmes at the market place.

For the evaluation of the programmes, changes in health status and health-relevant behaviour of the women as well as the costs of all phases of the exercise programmes and changes in health care utilisation were measured. Medical and sport scientists estimated the intervention as not invasive for the participants. Medical parameters were examined during the regular consultations of general practitioners by the study participants. Therefore, the study leader did not apply for an approval of the study by the responsible Ethics Committee.

However, in this paper, only the costing dimension of this physical activity programme will be analysed and discussed with reference to the “health asset concept” and the “participatory approach” to programme development and according to the cost dimension of the conceptual framework for economic evaluations for physical activity programmes [32, 36].

### 2.2. Programme Setup

The project was divided into five phases: (1) assessment; (2) design of the preventive intervention programmes; (3) programme implementation; (4) programme optimisation; (5) dissemination of the preventive programmes.

In the first phase—the phase of health asset assessment—options, chances and resources within the three settings were identified according to the health assets concept of the WHO. Therefore, in the first phase, the tasks of the project were to make contact with project partners and decision makers and to conduct face-to-face interviews with women in the target groups (duration of phase 1: six months).

In the second phase—the design of the preventive intervention programmes—project partners, representatives of the target group, stakeholders in the related political field and scientific experts took part in establishing setting-specific planning groups as well as an overall joint group. In the setting and joint groups, the programmes were developed in accordance with the participatory approach of the WHO (duration of phase 2: six months).

During the third phase, the developed physical activity programmes were implemented and scientifically evaluated (duration of phase 3: twelve months).

The fourth phase—optimisation of the programme—aimed at improving the established programmes and initiated the first steps in the rollout of these programmes to other regions of Germany in order to expand these preventive intervention programmes beyond the end of the project (duration of phase 4: six months).

During the fifth phase—the phase of dissemination—the conditions for successful project transfer to other regions were to be created (duration of phase 5: six months).

### 2.3. Costing

In terms of assessing the costs and the cost-effectiveness of the physical activity intervention programme, only the first, second, and third phases are relevant for the health economic evaluation. Note, however, that subject of costing of the third phase of the project was only activities related to the implementation of the health promotion interventions, whereas resource consumption and costs related to the scientific evaluation of the effects of the project were not included in the cost analysis. Also excluded from costing were the last two phases of the project as the main focus of these phases was to publish, promote, and disseminate the programme.

As stated above, one purpose of the study was to answer the question of whether the costs of the health assets assessment and the participatory project development should be included in evaluating the programme costs. Therefore, a straightforward implication for the cost analysis is a careful splitting into these project phases of “asset assessment,” “programme design,” and “programme implementation,” thus taking into account the specific requirements of the health asset concept and the participatory approach. The cost measurement of this intervention is based on the development of a detailed cost inventory comprising all relevant cost categories. All these cost categories were assigned to their pertinent costing dimension in a conceptual framework for the economic evaluation of physical activity programmes [32, 36]. In general, the cost dimension of the framework consists of the programme development costs and programme implementation costs. The costs of the health asset assessment phase include a part of the programme development costs comprising the personnel and nonpersonnel costs of the scientists leading the assessment. During the design phase, another part of programme development cost in terms of personnel and nonpersonnel costs of the scientists, the stakeholders and representatives of the target group arose. During the implementation phase of the intervention programme, cost components such as the implementation costs including personnel and nonpersonnel costs of the scientists, stakeholders and members of the target group for implementation, organisation of the physical activities programmes and recruiting participants as well as the costs of running the programme occurred. The recruiting costs include the costs that are connected with the recruitment of the participants to the physical exercise programmes. Information and other marketing activities as well as costs of the pilot workout and health seminar are regarded in the context of the health economic evaluation as activities of recruiting. And finally, the costs of the programme itself include costs of personnel and nonpersonnel costs for the accomplishment of the programmes, participant time costs, and the costs of supervising women's children during the courses. The components of costs to be considered in each phase and according to the cost dimension of the conceptual framework are shown in \.

All quantities of resources consumed were assessed retrospectively by questionnaires and personnel telephone interviews for each setting separately. Stakeholders, trainers, members of the target group, and scientists were interviewed to get all utilisation and cost data.

All resources were valued using market prices or—if market prices were not available—using values derived from cost manuals in Euros and adjusted to the year 2005. Actual and imputed costs of resource use and (non-) personnel costs were calculated and shown for a physical activity programme lasting for three months.

Resources utilized by a productive activity may include fixed assets which by definition are not fully consumed during their short-term use. The resources used in the project under study include two types of these fixed assets: gyms and sport equipment. For both types, two different approaches were applied to calculate their costs. For using the gyms for the physical activity programme, first, the actual rents to be paid by the programme organisers were used; this approach would be appropriate for programme costing from an organiser's or programme payer's view. Second, the imputed rent of a new gym was calculated following the customary capital costs approach; the corresponding cost values would be appropriate for assessing the costs in a societal perspective. The details of calculating the imputed capital costs of the use of gyms are given in Table 2. The resulting unit cost estimate per square metre and hour has been used to calculate the total costs of utilizing the gyms for the two programmes in the three different settings.

Similarly, for the equipment used in the physical activity programmes, cost values based on actual expenses and imputed costs were calculated. The calculation of imputed costs was based on the costs of new equipment, which would be needed according to the recommended manual for these physical activity programmes. Apart from the stereo equipment, all equipment needs are defined as needs per capita of women attending the programmes. These costs were valued with market prices for the year 2005 and amortized over the years of possible usage. The total equipment costs are calculated as costs per person for all types of equipment (except for the stereo system) times the number of participants plus the costs of stereo equipment. Actual equipment expenses were assessed by questionnaire from the programme organisers of each setting from the joint group.

Personnel time costs of the scientists, which include, for example, interviews with members of the target group, identification of the assets and contact with decision makers, were valued by typical gross earnings of scientists as public sector employees [37]. Time costs of representatives, for example time costs of attending the various meetings were valued by taking mean wage data of the lower social class (setting group) or by taking the mean wage rate of the German population (joint group) based on data published by the Federal Statistical Office of Germany [38]. For calculating the personnel costs of exercise instructors, again two different methods were applied. First, the number of course hours was valued with the contracted compensation per course hour or the appropriate wage rate. Second, the number of course hours plus the additional time investment (e.g., for pre- and post-presence and preparation) was valued with the corresponding unit cost figure.

From a societal perspective, the valuation of the time costs of the participants who take part in physical exercise programmes should be based on the principle of opportunity cost. The valuation of these costs depends on whether the time occupied in physical exercise replaces leisure or labour time (including housekeeping and unpaid activities) and whether the participants regard the exercise itself as a leisure activity. In this study, the time consumed for participating in the programme courses was assumed to be substituted for another type of leisure activity. Hence, the time cost for physical activity was valued at zero costs.

### 2.4. Sensitivity Analysis

A sensitivity analysis was conducted to determine how “sensitively” the results of the cost analysis react to changes in the values of single-cost parameters. Several assumptions were varied, such as the amortisation of equipment, nonpersonnel and personnel costs. The average usage of a single piece of equipment was based on personnel information from a professional sports scientist. Variation in the usage of a single item cannot be excluded; therefore, ±1 year of amortisation of equipment was calculated. Additionally, ±50% of all nonpersonnel cost positions was calculated to reflect the uncertainty of the chosen market prices. Furthermore, the setting and the joint group are made up of different representatives who would incur different personnel time costs according to their work. Thus, ±10% of the average wage rate is added and subtracted to approximate the real average wage rate. The costs of exercise instructors were also calculated from the programme payer's perspective taking the actual wage rate and, for the societal perspective, calculating the additional time of pre- and post-preparation of the physical activity programme according to the questionnaire filled out retrospectively by trainers in the project. The personnel costs of trainers and child care were also calculated within ±10% to account for the uncertainty of the given hourly wage rate.

All costs were calculated and presented as minimum (min) costs, maximum (max) costs and personal information (PI) costs. The actual costs from the programme payer's perspective and the societal perspective (including imputed costs of the gym, equipment, and exercise instructors instead of the corresponding financial values) are calculated and reported separately.

## 3. Results

The results of our various cost calculations are shown in detail in different perspectives and partitions in Tables 3–7. First of all, in Table 3, the results of calculating the imputed costs of equipment and the results of the corresponding sensitivity analysis (by varying the working life of the equipment) are reported. In the baseline assessment, the total equipment costs over the three settings added up to 284.9 € for the low-intensity programme, 619.0 € for the high-intensity programme, and 903.9 € for both programmes.

Table 4 presents the results of the detailed calculation of costs accruing during the three phases of programme including all types of inputs except for the services provided to the project by the team of scientists. The table shows the resources and the corresponding cost figures by setting, types of activity, and various categories of inputs and costs. Note that for three cost items (costs of gym, equipment, and instructors) two different cost items—one based on financial data and one based on imputed cost data—have been calculated in order to allow for different costing perspectives (payer's versus societal perspective).

Next, Table 5 provides the resource and cost figures related to the project-assisting activities (except for evaluation research activities) of the team of scientists by the three settings and the three project phases. As can be seen from the table, in all three phases substantial additional inputs into the project have been made by the scientific team in addition to its evaluation research activities.

In Table 6, the results of the cost calculations are summarised. Costs from the programme payer's and societal perspective including the results of the sensitivity analyses are shown and categorised according to the study phases and, as far as possible, according to the cost categories of the conceptual framework.

The total costs the project amounted to 128,353 € if the payers' perspective is chosen, and 133,074 € in a societal perspective. These figures can be subdivided into costs of 35,618 € for the asset assessment, 48,789 € for the programme design (which in turn add up to programme development costs of totally 84,407 €), and costs of 43,945 € (payer's perspective) and 48,666 € (societal perspective), respectively, for programme implementation activities. Hence, programme implementation costs accounted only for 34% (payer's perspective) and 37% (societal perspective), respectively, of total project costs. Focussing exclusively on the running costs of the programmes, the corresponding percentages were 3% (payer's perspective) and 6% (societal perspective), respectively. However, one should take into account that the predominant part of programme costs represents variable costs with more or less constant costs per participant so that larger numbers of participants would mean correspondingly higher costs, whereas, the predominant part of residual programme implementation costs and actually all costs accruing in the asset assessment phase and the programme design phase, in contrast, represent fixed costs. Therefore, the proportion of programme costs of total implementation costs and total project costs may well rise with an increasing number of women participating in the physical activity programmes and/or with continuing the programmes in the future.

Finally, Table 7 shows cost figures related to various combinations of project phases. Adding the costs of the assessment phase to the costs of the implementation phase, the total costs come to €79,563 (62% of the total costs) from the programme payer's perspective and €84,284 from the societal perspective. The costs for developing the programme design plus the costs of programme implementation add up to €92,735 (72% of the total costs) from the programme payer's perspective or €97,456 from the societal perspective. Adding on top of that the costs of the design phase, the total costs amount to €128,353 or €133,074, respectively, as already mentioned above. These combined costs can be considered as cost figures representing different decision contexts which will be discussed below.

## 4. Discussion

Dependent on the chosen perspective and the scope of costing, total costs varied substantially. The appropriate scope of costing depends on the type of decision that has to be made. Four typical decisions contexts should be distinguished (see also superscript numbers in Table 7):

1. if the decision is whether or not the implemented physical activity programmes should be put into practice in other similar settings or be continued in the future, only the costs of the “programme implementation phase” should be taken into consideration;
2. the condition precedent to the second decision is the identification and assessment of the assets and the implementation of equally effective health promotion programmes in other places or settings. This decision comprises the costs of the “asset assessment phase” plus the costs of the “programme implementation phase;”
3. the third type of a decision context relates to a situation in which the intervention programme has to be redesigned and implemented in similar settings. This decision includes the costs of the “programme design phase” plus the costs of the “programme implementation phase;”
4. the fourth decision can be differentiated into two alternatives. The first is the identification and assessment of the asset, programme design and programme implementation of adapted health promotion programmes in different settings on account of the decision in favour of a participatory project development approach. The second alternative involves the application of the full health assets approach in other places or settings in order to develop new health promotion programmes for the targeted population. Both decisions include all the costs of all phases, the health asset assessment phase plus the programme design phase plus the programme implementation phase.

The scope of cost components included in the analysis and the identification of the decision context clearly affect the cost-effectiveness of the implemented health promotion programmes. In consequence, the incremental cost-effectiveness ratio of the programme will be rather sensitive with regard to a variation in the scope of costing. Moreover, a longer duration of the intervention programme would amortise the costs of health asset assessment and programme design, and/or the transferability of the intervention programme to other regions would allow an allocation of costs to a larger number of participants.

The main limitations of this cost analysis could be found in the valuation of the data and the data collection. The utilisation of physical units and productivity were monetized using standard values or average gross wage. This could result in limited precision for total costs; an under- or over-estimation cannot be excluded. Quantities of resources were assessed by participants, scientists, stakeholders and sports trainers using a self-administered, retrospective, standardised questionnaire and interview. A bias cannot be excluded as well as a recall of the utilisation retrospectively which cannot eliminate inaccuracy in the self-reported data.

## 5. Conclusion

Dependent on the scope of costing and the chosen perspective, total programme costs varied substantially. From the programme payer's (societal) perspective, the costs of programme implementation amounted to €43,900 (€48,700). Adding the costs for developing the programme design of €48,800 results in total costs of €92,700 (€97,500); adding on top of that the costs of asset assessment running to €35,600 would total €128,300 (€133,100). Interestingly, programme implementation costs including recruitment costs make up only 34% to 37% of total costs, dependent on the chosen perspective of analysis.

The findings of this study demonstrate that the results of an economic evaluation of a physical activity programme as part of health promotion efforts according to the WHO health asset approach will be highly sensitive to whether or not and to what extent programme development costs are included in the cost calculations. The clarification of this open methodological question is of the utmost importance for the economic evaluation of health promotion activities based on the health asset concept and using a participatory approach to programme development.

Therefore, the identification of the decision context that is to be supported by the evaluation as well as the assumptions made in terms of the generalizability of the evaluation results is of crucial importance for the validity of an economic programme evaluation.

This study did not answer the question of the cost dimension of an all-encompassing intervention programme. But, finally, this study addresses and invites decision makers to consider these cost dimensions with regard to the willingness-to-pay trade-off and the transferability of the results. Further economic evaluations of effective primary prevention programmes must be performed, and utilisations and costs have to be assessed in detail so that health care decisions may be improved in the future.

## Figures and Tables

**Table 1:** Input-oriented subdivision of costs by project phases.

(I) Health asset assessment phase (6 months)/programme development costs	

Personnel costs of the scientists (e.g., time costs of workshops, meetings and interviews with members of the target group, identification of the assets, contact with decision makers, etc.)	
Nonpersonnel costs (e.g., travelling)	

(II) Programme design phase (6 months)/programme development costs	

Personnel costs of the scientists, the stakeholders and representatives of the target group (e.g., time costs of meetings and workshops)	
Nonpersonnel costs (e.g., travelling)	

(III) Programme implementation phase (12 months)/programme implementation costs	

Implementation costs: personnel costs of the scientists, the stakeholders, and of some members of the target group (acquisition and recruitment of participants, implementation, organisation and administration of the programmes, pilot workout, and health seminar)	
Implementation costs: nonpersonnel costs (material costs)	
Recruitment costs: costs of marketing and information (e.g., posters and flyers)	
Recruitment costs: costs of pilot workout and health seminar (rent of the gym or related infrastructure, trainer or health specialist, costs of child care of participant's children, and sports equipment) and participant time costs	
Programme costs: costs of infrastructure, costs of the physical exercise programmes (rent of the gym or related infrastructure, trainer including pre- and post-preparation, and sports equipment), costs of child care of participant's children, and participant time costs	

**Table 2:** Imputed capital costs of the gym.

	Construction costs for a gym in the year 1998 (15*27 m²)		920,325 €	2,272 €
(1)	Adjusted for the year 2005 (by construction price index)	*1.0502		2,386 €
(2)	Linear amortisation p.a., 30 years	/30		80 €
	Average fixed capital	(1)/2		1,193 €
(3)	Interest expense rate of 3%	*0.03		36 €
(4)	Capital costs per m² p.a.	(2) + (3)		115 €
	Current costs p.a.		76,694 €	189 €
(5)	Adjusted for the year 2005 (by consumer price index)	*1.1051		209 €
	Costs of gym per m² p.a.	(4) + (5)		289 €
	Number of hours for utilisation (8 × 365)		2,920	
	Costs per m² and hour	**0.1112** **€**		

Abbreviations: p.a.: per annum; m^2^: square metre.

**Table 3:** Per capita and total imputed costs of equipment by type of equipment, type of programme, and duration of working life.

Required materials	Acquisition costs	Amortisation of years			Amortised costs		
		Ø	min.	max.	Ø	min.	max.
Low-intensity programme							

Mat	21.4 €	3	2	4	1.8 €	1.3 €	2.7 €
Swiss ball	6.9 €	6	5	7	0.3 €	0.2 €	0.3 €
Reflex massage ball	5.6 €	6	5	7	0.2 €	0.2 €	0.3 €
Tennis ball	1.8 €	2	1	3	0.2 €	0.1 €	0.4 €
Thera-Band	10.9 €	2	1	3	1.4 €	0.9 €	2.7 €
Softball	7.5 €	6	5	7	0.3 €	0.3 €	0.4 €
Volleyball	28.0 €	2	1	3	3.5 €	2.3 €	7.0 €
Tennis ring	4.9 €	10	9	11	0.1 €	0.1 €	0.1 €
Little sack	8.8 €	3	2	4	0.7 €	0.6 €	1.1 €
**Total costs/person**	**95.8 €**				**8.6 €**	**6.1** **€**	**15.1** **€**
Stereo equipment	100.0 €	10	9	11	2.5 €	2.3 €	2.8 €

Setting	No. of participants						

Sports club	8				68.5 €	51.1 €	123.4 €
Company	8				68.5 €	51.1 €	123.4 €
Residential district	17				148.0 €	106.0 €	259.1 €
**Total**	** 33**				**284.9 €**	**208.2 €**	**505.9 €**

High-intensity programme							

Mat	21.4 €	3	2	4	1.8 €	1.3 €	2.7 €
Swiss ball	6.9 €	6	5	7	0.3 €	0.2 €	0.3 €
Reflex massage ball	5.6 €	6	5	7	0.2 €	0.2 €	0.3 €
Tennis ball	1.8 €	2	1	3	0.2 €	0.1 €	0.4 €
Thera-Band	10.9 €	2	1	3	1.4 €	0.9 €	2.7 €
Softball	7.5 €	6	5	7	0.3 €	0.3 €	0.4 €
Volleyball	28.0 €	2	1	3	3.5 €	2.3 €	7.0 €
Tennis ring	4.9 €	10	9	11	0.1 €	0.1 €	0.1 €
Little sack	8.8 €	3	2	4	0.7 €	0.6 €	1.1 €
Small dumbbells	6.5 €	10	9	11	0.2 €	0.1 €	0.2 €
Water-polo ball	5.0 €	2	1	3	0.6 €	0.4 €	1.2 €
Hopping ball	3.0 €	10	9	11	0.1 €	0.1 €	0.1 €
Medicine ball	22.0 €	10	9	11	0.6 €	0.5 €	0.6 €
Cloth	6.9 €	3	2	4	0.6 €	0.4 €	0.9 €
Rope	9.5 €	3	2	4	0.8 €	0.6 €	1.2 €
**Total costs/person**	**148.6 €**				**11.3 €**	**8.3 €**	**19.2 €**
Stereo equipment	100.0 €	10	9	11	2.5 €	2.3 €	2.8 €

Setting	No. of participants						

Sports club	10				115.7 €	84.8 €	195.1 €
Company	8				93.1 €	68.3 €	156.7 €
Residential district	36				410.2 €	299.4 €	695.2 €

**Total **	** 54**				**619.0 €**	**452.4 €**	**1,047.0 €**

Abbreviations: no.: number; max.: maximum; min.: minimum; PI: personnel information.

**Table 4:** Resource use and total cost of programme implementation (without inputs provided by the scientific team) by setting, types of activity, and input/cost categories.

		Sports club			Company			Residential district		
		PI	min.	max.	PI	min.	max.	PI	min.	max.
Nonpersonnel costs										

Costs of infrastructure and equipment										

Floor area of gym (m²)	HI	350			70			300		
	LI	150			70			300		
Duration of an exercise unit (minutes)	HI	90			90			90		
	LI	60			60			60		

**Total costs of gym**		**120 €**	**60 €**	**180 €**	**0 €**	**0 €**	**0 €**	**12 €**	**6 €**	**18 €**
*Total imputed costs of gym*		*900 €*	*450 €*	*1,351 €*	*233 €*	*117 €*	*350 €*	*1,001 €*	*500 €*	*1,501 €*
**Total costs of equipment**		**600 €**	**300 €**	**900 €**	**0 €**	**0 €**	**0 €**	**100 €**	**50 €**	**150 €**
*Total imputed costs of equipment*		*184 €*	*136 €*	*319 €*	*162 €*	*119 €*	*280 €*	*558 €*	*405 €*	*954 €*

Costs of marketing										

Concept/advertisement		577 €	288 €	865 €	none	none	none	232 €	116 €	348 €
Printing costs (posters)		incl.	incl.	incl.	none	none	none	60 €	30 €	90 €
Printing costs (flyers)		none	none	none	none	none	none	156 €	78 €	234 €
Announcement		none	none	none	none	none	none	none	none	none
Miscellaneous costs		50 €	25 €	75 €	none	none	none	none	none	none
**Total costs of marketing**		**627 €**	**313 €**	**940 €**	**0 €**	**0 €**	**0 €**	**448 €**	**224 €**	**672 €**

Costs of pilot workout or 3-day health seminar										

		Pilot workout 3*3 h			3-day health seminar			2*pilot workout 3*3 h		
Costs for the trainer and/or the organiser of the course		207 €	186 €	228 €	1,221 €	1,099 €	1,343 €	414 €	373 €	455 €
Costs of child care		90 €	81 €	99 €	none	none	none	180 €	162 €	198 €
Rent for rooms		none	none	none	none	none	none	none	none	none
Material costs		none	none	none	none	none	none	none	none	none
Other (e.g., Catering)		none	none	none	480 €	240 €	720 €	none	none	none
**Total costs of pwo and hs**		**297 €**	**267 €**	**327 €**	**1,701 €**	**1,339 €**	**2,063 €**	**594 €**	**535 €**	**653 €**

Personnel costs										

Participant time costs		0 €	0 €	0 €	0 €	0 €	0 €	0 €	0 €	0 €

Costs of exercise instructors and childcare (for a 3-month exercise programme)										

Wage per hour of instructors		23 €	21 €	25 €	31 €	28 €	34 €	20 €	18 €	22 €
**Total costs of instructors **		**690 €**	**621 €**	**759 €**	**930 €**	**837 €**	**1,023 €**	**600 €**	**540 €**	**660 €**

Mean additional time effort of the trainer										

Oneway (minutes)	HI	5			20			15		
	LI	4			20			15		
Presence (before and after) (minutes)	HI	18			15			20		
	LI	25			15			20		
Preparation (minutes)	HI	20			20			30		
	LI	20			20			30		

*Total imputed costs of instructors (inclusive additional time)*		*1,242 €*	*1,118 €*	*1,366 €*	*2,093 €*	*1,883 €*	*2,302 €*	*1,400 €*	*1,260 €*	*1,540 €*
Costs of child care per hour		8 €	7 €	9 €	none	none	none	8 €	7 €	9 €
**Total costs of child care **		**240 €**	**216 €**	**264 €**	**none**	**none**	**none**	**240 €**	**216 €**	**264 €**

Costs of temporal effort of the joint group (7 meetings)										

Ø number of persons/meeting*		5			7			7		
Conceptual design (minutes)		280			280			280		
**Total costs (Conceptual design)**		**446 €**	**401 €**	**490 €**	**624 €**	**562 €**	**687 €**	**624 €**	**562 €**	**687 €**
Organ./implementation (minutes)		200			200			200		
Costs of implementation		318 €	287 €	350 €	446 €	401 €	490 €	446 €	401 €	490 €
Organ./implementation of pilot workout (minutes)		50			50			50		
Costs (implementation of pilot workout)		80 €	72 €	88 €	111 €	100 €	123 €	111 €	100 €	123 €
Organ./implementation of exercise facilities (minutes)		65			65			65		
Costs (implementation of exercise facilities)		104 €	93 €	114 €	145 €	130 €	159 €	145 €	130 €	159 €
Total time (minutes/person)		595			595			595		
Total costs (implementation)		502 €	451 €	552 €	702 €	632 €	772 €	702 €	632 €	772 €

Personnel costs of temporal effort of the setting group (5 meetings)										

Ø number of persons/meeting*		12			12			12		
Conceptual design (minutes)		197			263			300		
**Total costs (conceptual design)**		**394 €**	**355 €**	**433 €**	**526 €**	**473 €**	**579 €**	**600 €**	**540 €**	**660 €**
Organ./implementation (minutes)		177			263			270		
Costs of implementation		354 €	319 €	389 €	526 €	473 €	579 €	540 €	486 €	594 €
Organ./implementation of pilot workout (minutes)		89			118			135		
Costs (implementation of pilot workout)		178 €	160 €	196 €	236 €	212 €	260 €	270 €	243 €	297 €
Organ./implementation of exercise facilities (minutes)		89			118			135		
Costs (implementation of exercise facilities)		178 €	160 €	196 €	236 €	212 €	260 €	270 €	243 €	297 €
Total time (minutes/person)		552			762			840		
**Total costs (implementation)**		**710 €**	**639 €**	**781 €**	**998 €**	**898 €**	**1,098 €**	**1,080 €**	**972 €**	**1,188 €**

Abbreviations: h: hour; hs: health seminar; m: metre; max: maximum; min.: minimum; PI: personnel information; organ.: organisation; pwo: pilot workout.

Italic figures: imputed cost values (as values alternative to financial values).

*Without scientists.

**Table 5:** Working time and total personnel and nonpersonnel costs of scientists by setting and project phase.

Setting	Asset assessment phase	Programme design phase	Programme implementation phase	**Total**
Sports club: weeks				
(scientists/students)	3/0.5	8/0.5	8/4	
Personnel costs	3,905 €	10,317 €	10,721 €	**24,943** **€ **
Nonpersonnel costs	700 €	900 €	1,250 €	**2,850** **€**

Company: weeks				
(scientists/students)	2/0.5	8/0.5	4/0.5	
Personnel costs	2,623 €	10,317 €	5,187 €	**18,127** **€ **
Nonpersonnel costs	500 €	500 €	50 €	**1,050** **€**

Residential district: weeks				
(scientists/students)	3/0.5	12/0.5	5/4	
Personnel costs	3,905 €	15,446 €	6,874 €	**26,225** **€ **
Nonpersonnel costs	1,250 €	2,000 €	1,250 €	**4,500** **€**

Miscellaneous: weeks				
(scientists/students)	15/0	5/0.5	5/0.5	
Personnel costs	19,236 €	6,470 €	6,470 €	**32,175** **€ **
Nonpersonnel costs	3,500 €	250 €	250 €	**4,000** **€**

** Total**	** 35,618** **€**	** 46,199** **€**	** 32,052** **€**	** 113,870** **€**

**Table 6:** Total project costs by project phases and input-oriented cost categories.

	PI	min.	max.
(I) Asset assessment phase (6 months)/programme development costs			

Personnel costs of the scientists	29,668 €	26,701 €	32,635 €
Nonpersonnel costs	5,950 €	2,975 €	8,925 €
Total	35,618 €	29,676 €	41,560 €

(II) Programme design phase (6 months)/programme development costs			

Personnel costs of the scientists, the stakeholders and representatives of the target group	45,139 €	40,626 €	49,653 €
Nonpersonnel costs	3,650 €	1,825 €	5,475 €
Total	48,789 €	42,451 €	55,128 €

(III) Programme implementation phase (12 months)/programme implementation costs			

Implementation costs: personnel costs of the scientists, the stakeholders and of some members of the target group	33,947 €	30,552 €	37,341 €
Implementation costs: nonpersonnel costs	2,800 €	1,400 €	4,200 €
Recruitment costs: costs of marketing and information	1,075 €	537 €	1,612 €
Recruitment costs: costs of pilot workout and health seminar	2,592 €	2,141 €	3,043 €
Programme costs (actual costs)	3,532 €	2,846 €	4,218 €
*Programme costs (imputed costs)*	*8,253 €*	*6,421 €*	*10,491 €*
Total			
Payers perspective	43,946 €	37,446 €	50,414 €
*Societal perspective *	*48,666 €*	*41,021 €*	*56,687 €*

All phases (I–III)			

Payer's perspective	128.353 €	109,603 €	147,102 €
*Societal perspective*	*133,074 €*	*113,178* €	*153,375 €*

Abbreviations: max.: maximum; min.: minimum; PI: personnel information.

Italic figures: imputed cost values (as values alternative to financial data).

**Table 7:** Summary of the costs (€) of the physical activity programme differentiated in the special decision context and perspective.

Project phase	Cost category	PI	min.	max.
(I) asset assessment	Programme development costs	35,618 €	29,676 €	41,560 €
(II) programme design	Programme development costs	48,789 €	42,451 €	55,128 €

Programme payer perspective				

(III) programme implementation^(1)^	Implementation costs, recruitment costs, programme costs, participant time costs	43,945 €	37,476 €	50,415 €
(I+ III) (asset assessment + programme implementation)^(2)^	Development costs (I), implementation costs, recruitment costs, programme costs, participant time costs	79,563 €	67,152 €	91,974 €
(II+III) (programme design + programme implementation)^(3)^	Development costs (II), implementation costs, recruitment costs, programme costs, participant time costs	92,735 €	79,927 €	105,543 €
(I+II+III)^(4)^	Total	128,353 €	109,603 €	147,103 €

Societal perspective				

(III) programme implementation^(1)^	Implementation costs, recruitment costs, programme costs, participant time costs	48,666 €	41,051 €	56,687 €
(I+ III) (asset assessment + programme implementation)^(2)^	Development costs (I), implementation costs, recruitment costs, programme costs, participant time costs	84,284 €	70,727 €	98,247 €
(II+III) (programme design + programme implementation)^(3)^	Development costs (II), implementation costs, recruitment costs, programme costs, participant time costs	97,456 €	83,502 €	111,816 €
(I+II+III)^(4)^	Total	133,074 €	113,178 €	153,375 €

^(1–4)^decision (see Discussion).

Abbreviations: PI: personnel information; min.: minimum; max.: maximum.
